# Akinetic mutism reversed by inferior parietal lobule repetitive theta burst stimulation: Can we restore default mode network function for therapeutic benefit?

**DOI:** 10.1002/brb3.2180

**Published:** 2021-06-17

**Authors:** Tressie M. Stephens, Isabella M. Young, Christen M. O'Neal, Nicholas B. Dadario, Robert G. Briggs, Charles Teo, Michael E. Sughrue

**Affiliations:** ^1^ Department of Neurosurgery University of Oklahoma Health Sciences Center Oklahoma City Japan; ^2^ Cingulum Health Sydney NSW Australia; ^3^ Rutgers Robert Johnson Wood School of Medicine New Brunswick NJ USA; ^4^ Centre for Minimally Invasive Neurosurgery Prince of Wales Private Hospital Sydney NSW Australia

**Keywords:** default mode network, glioblastoma, right frontal tumor, theta burst stimulation, transcranial magnetic stimulation, tumor resection

## Abstract

**Background:**

Transcranial magnetic stimulation is a noninvasive treatment used to modulate cortical excitability. Its use over the last two decades has expanded, ranging from psychiatric disorders to traumatic brain injury and poststroke rehabilitation.

**Objectives:**

We present the case of a 59‐year‐old male patient who presented in a decreased state of consciousness due to a right frontal glioblastoma, wherein his state was not improved by a successful surgery and could not be explained by any other condition. Due to his poor prognosis, we examine the benefits of receiving transcranial magnetic stimulation treatment to improve his akinetic mutism.

**Methods:**

We utilized independent component analysis with resting‐state functional magnetic resonance imaging (rsfMRI) to better understand his cortical functionality. The imaging suggested absence of the default mode network (DMN). The patient underwent five sessions of navigated intermittent theta burst stimulation to the ipsilesional inferior parietal lobule and inferior frontal gyrus, with the aim of improving his default mode network functionality.

**Results:**

No other treatments resulted in an improvement of this patient's condition; however, 3 weeks following transcranial magnetic stimulation treatment, the patient was more alert and interactive, and his follow‐up rsfMRI scan demonstrated a partially intact default mode network.

**Conclusion:**

This case raises important questions regarding the clinical utility of transcranial magnetic stimulation to improve the connectivity of important cerebral networks and subsequent related functional recovery.

## Graphical abstract

We present the case of a 59‐year‐old male patient who presented in a decreased state of consciousness due to a right frontal glioblastoma, wherein his state was not improved by a successful surgery and could not be explained by any other condition. Due to his poor prognosis, we utilized independent component analysis with resting state functional magnetic resonance imaging to better understand his cortical functionality which suggested an absence of a default mode network. The patient underwent five sessions of navigated intermittent theta burst stimulation to the ipsilesional inferior parietal lobule and inferior frontal gyrus, with the aim of improving his default mode network functionality, within 3 weeks of treatment, the patient was more alert and interactive, and his follow‐up scan demonstrated a partially intact default mode network.

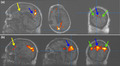

## 1 INTRODUCTION

Injury to the reticular activating system is the best‐known cause of unresponsiveness, and often one of the most severe, but it is by no means is the only mechanism by which people can remain in a persistently poor state of alertness and with a limited ability to interact with their surroundings (Hannawi et al., 2015). Injuries to the thalamus, basal ganglia, basal forebrain, and medial frontal lobe can all yield distinct or overlapping neurologic conditions characterized by some combination of inability to follow commands, mutism, abulia, lack of initiation, and failure to regard or track in their surroundings (Briggs et al., 2021; Hannawi et al., 2015). Regardless of the mechanism, poor neurologic condition is a risk factor for numerous other medical complications and poor alertness and ability to interact not only limits daily functional capacity, but also renders rehabilitation impossible.

Unfortunately, in essentially the entire history of the clinical practice of neurology and neurosurgery, there have been few direct therapies for unresponsiveness. The principle foundation of most therapeutic approaches has been avoidance, for example, to prevent transtentorial herniation and damage to the reticular activating system (Hannawi et al., 2015). For those unfortunate patients in whom avoidance is no longer clinically feasible, the therapeutic strategies widely practiced focus on supportive care to alleviate symptom burden (Ebke et al., 2018; Padilla & Domina, 2016; Threlkeld et al., 2018). While preventing intracranial pressure elevation, supporting ventilation and metabolic demands, and treating problems related to immobility can facilitate some impressive recoveries in patients with severe illnesses, the majority of patients do not recover (Padilla & Domina, 2016; Threlkeld et al., 2018). Thus, alternative neurorehabilitation strategies are needed in these nonresponsive patients to improve functional capacity, vocational abilities, and neurocognition.

In this report, we present our experience with a patient who presented as an akinetic mute from a large right medial frontal glioma who remained chronically unresponsive after surgery. When we found that he lacked evidence of an independent functional component containing the default mode network (DMN), we utilized the novel approach of applying repetitive intermittent theta burst stimulation (iTBS) as an off‐label transcranial magnetic stimulation (TMS) therapy to attempt to stimulate the DMN (Cardenas‐Morales et al., 2011; Shang et al., 2019). This treatment demonstrated striking results, and the evidence that the DMN connectivity was restored raises the interesting hypothesis that targeted TMS can serve as an active form of acute inpatient rehab and that the chronic loss of DMN connectivity is a treatable medical problem.

## 2 METHODS

This study was performed with approval from our institutional review board (IRB #3199).

### 2.1 MRI acquisition for image‐guided targeting

Magnetic resonance imaging (MRI) was performed on a 1.5 T GE whole‐body scanner (Thomason et al., 2011). During the resting‐state experiment, the patient completed an 8‐min scan during which they were instructed to lay still with their eyes closed. All resting‐state functional MRI (rsfMRI) scans were conducted following the anatomical localizer, a field inhomogeneity shim, and a 4‐min perfusion scan.

For this study, 29 axial slices were acquired with 4 mm slice thickness (no skip) (Thomason et al., 2011). A T2*‐sensitive gradient echo spiral in/out pulse sequence was used for all rfMRI imaging (TR = 2,000 ms, TE = 30 ms, flip angle = 77°, FOV = 22 cm, 64 × 64) (Glover, 2012). An automated high‐order shimming procedure, based on spiral acquisitions, was used to reduce B0 heterogeneity as spiral in/out methods have been shown to increase signal‐to‐noise ratio and BOLD contrast‐to‐noise ratio and also to reduce signal loss in regions compromised by susceptibility‐induced field gradients generated near air‐tissue interfaces, such as the prefrontal cortex (Glover & Law, 2001; Kim et al., 2002). A high‐resolution volume scan (140 slices, 1 mm slice thickness) was collected for the patient using a spoiled gradient‐recalled sequence for T1 contrast (TR = 3,000 ms, TE = 68 ms, TI = 500 ms, flip angle = 11°, FOV = 25 cm, 256 × 256). During the resting‐state scan, the patient's heartrate and respiration waveform were recorded.

### 2.2 Independent component analysis and identification of cortical networks

The functional data were preprocessed using the Multivariate Exploratory Linear Decomposition into Independent Components version 5.0, part of FSL toolbox (Storti et al., 2013). The images were smoothed with a Gaussian kernel of full width at half‐maximum of 8 mm. A slice timing correction was used to correct for the different acquisition times. The data were then preprocessed with high‐pass temporal filtering (cutoff of 100 s) and with the removal of nonbrain structures from the echo planar imaging volumes [Brain Extraction Tool]. Independent component analysis (ICA) threshold was set to 0.66 in order to eliminate extraneous noise from the brain networks. This analysis yielded a total of 40 independent components on the pre‐TMS scan and 48 post‐TMS.

All components were reviewed to identify distinct individual brain networks by examining ICA threshold maps, which were displayed on the patient's postoperative anatomical MRI brain using the Multi‐image Analysis GUI, Mango version 4.0.1 (http://ric.uthscsa.edu/mango/). We planned to identify the DMN based on previous literature and published functional activation maps (Kim et al., 2002; Smith et al., 2009; Storti et al., 2013), which suggest the DMN includes the medial prefrontal cortex, posterior cingulate/precuneus, posterior inferior parietal lobes, lateral temporal lobes and cortex, and hippocampal formation (Van Calster et al., 2017).

### 2.3 Transcranial magnetic stimulation

No independent components were found on the pre‐TMS ICA resembling the DMN. Therefore, following tumor resection, we targeted the right inferior parietal lobule using stereotactic neuro‐navigation. The patient's anatomical images were loaded for TMS planning on Localite TMS Navigator Version 3.0.48 (Localite TMS Navigator, Localite, Sankt Augustin, Germany) as a NIFTI file. Both the target and entry point were selected for navigation to allow accurate guidance of the coil and current due to abnormal, postresection anatomy.

TBS was administered with a Magventure MagproX100 with Magoption and a figure 8 coil, specifically Cool B‐65A/P coil system (Magventure Inc, Denmark) at 80% of resting motor threshold (RMT) for the patient. Motor threshold was found by stimulating the right primary motor cortex, with the lowest amplitude required to create a visible twitch at the left Flexor Digitorum Interossei and Abductor Pollicis Brevis in 5/10 trials. The patient received pulses applied at 50 Hz in three pulse bursts at a frequency of 5 Hz, with an intertrain interval of 8.0 s, all in accordance with standard iTBS protocols (Rossi et al., 2009).

## 3 CASE DESCRIPTION

**FIGURE 1:**
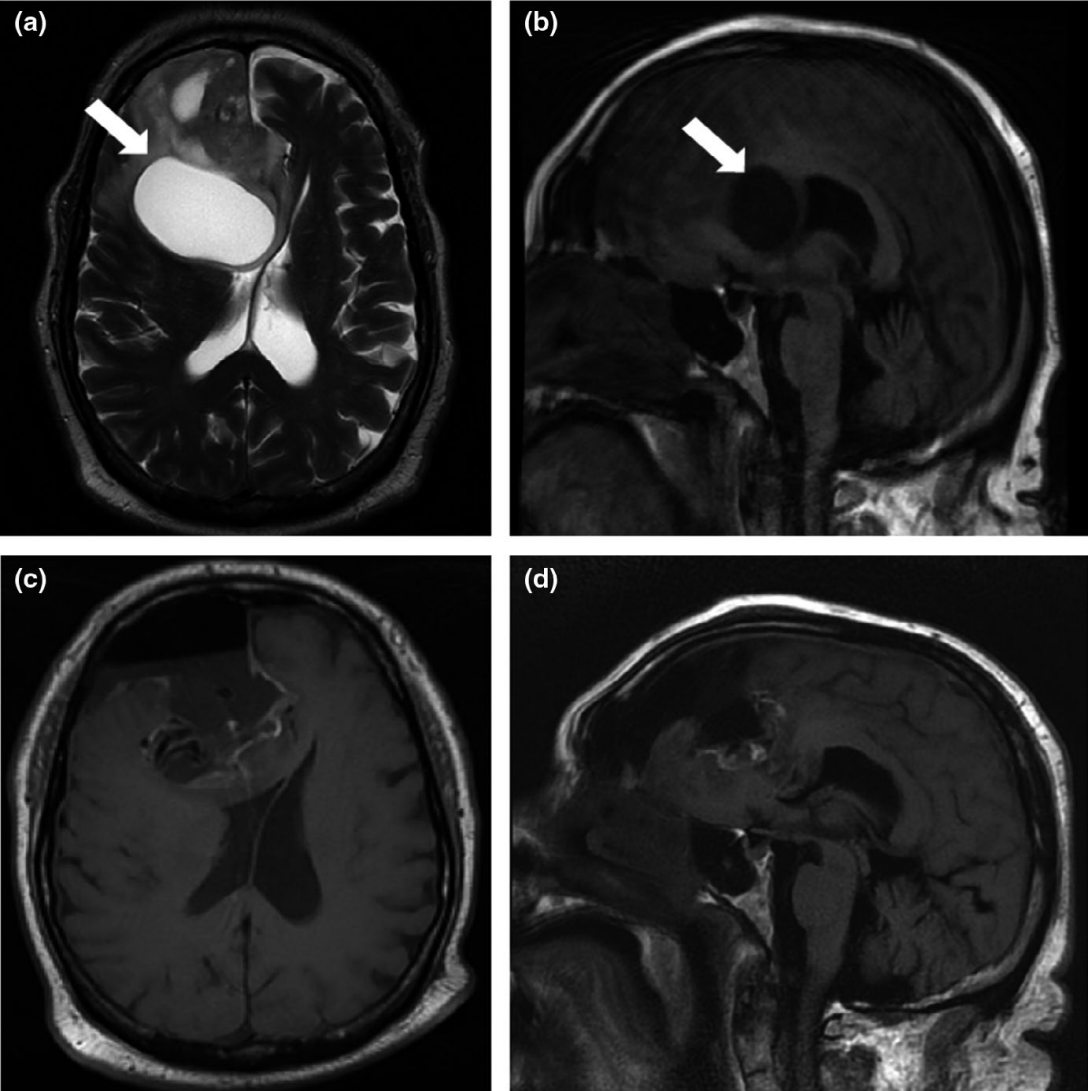
(a) T2‐weighted axial and (b) T1‐weighted sagittal preoperative magnetic resonance imaging demonstrates a large, cystic glioblastoma in the right frontal lobe that extends medially to compress midline structures. White arrows highlight the position of the cystic component of the tumor in panels (a) and (b). (c) T1‐weighted axial and (d) T1‐weighted sagittal imaging demonstrates the extent of tumor resection following awake craniotomy

The patient is a 59‐year‐old male with a large partially cystic right medial frontal glioblastoma multiforme (GBM) who presented to an outside hospital with 1‐month progressive altered mental status, syncope, and difficulty ambulating. By the time he presented to our care, he was noted to have limited functional mobility, minimal spontaneous movements, and was unable to talk or to follow commands. Preoperative imaging demonstrated the GBM's involvement of most of the medial frontal lobe, the anterior cingulate, and genu of the corpus callosum (Figure 1).

Following surgery, postoperative imaging was obtained for the patient, which demonstrated excellent tumor resection, with no evidence of the removal of the basal ganglia, ischemic stroke, or hematoma (Figure 1). Despite this, he remained in poor neurologic condition and required reintubation to protect his airway due to unresponsiveness. Eventually, he required a ventriculoperitoneal shunt placement and a percutaneous endoscopic gastronomy placement as he was unable to remain alert for feeding. The postoperative course was complicated by hydrocephalus, severely elevated intracranial pressure and dysphagia, requiring an external ventricular drain.

Seven weeks following surgery, he remained in as poor a neurologic condition as when he initially arrived, and it was determined that because he had made no progress. His Karnofsky performance status (KPS) was an abysmal score of 20, and he was not a candidate for adjuvant therapy for his glioma. We had repeatedly imaged him to show that his shunt appeared to be working, and there were no obvious other structural causes of his poor neurologic condition. Continuous electroencephalogram had been unrevealing. Repeated cerebrospinal fluid sampling had never shown any sign of infection. Multiple rounds of laboratory studies had shown no evidence of metabolic causes of his poor status such as hepatic dysfunction, electrolyte, thyroid, or adrenal disturbances. We had eliminated all sedating medicine. In short, there was no obvious confounding causes of his poor neurologic condition other than the injury caused to his frontal lobe by the tumor and its removal.

He had been waiting in the hospital for placement in a long‐term care facility for 2 weeks when we became curious about whether there was anything that could be done for him, so we obtained a rsfMRI image. When this was processed using ICA, we found that none of the components contained a set of activated areas suggestive of the DMN in either hemisphere. While ICA can sometimes split components, our experience with clinical grade images processed in this way is that the DMN is always present. For this reason, we hypothesized that loss of the anterior medial frontal component of the DMN had caused the loss of a coherent DMN bilaterally.

We discussed the idea of attempting to treat this with the family. More specifically, we discussed using repetitive TMS in an off‐label use to try to improve his neurologic function. We told them that this was a completely experimental use of the device; however, given that without neurologic improvement, his death was certain. We discussed that we had no other therapies to offer to try to make him better and that it is unlikely that the treatment would worsen his grim natural history. They were willing to try this treatment option.

The DMN classically is made up of three main areas per hemisphere (the medial frontal/anterior cingulate (ACC), the posterior cingulate (PCC), and the inferior parietal lobule (IPL)). Two of these are typically inaccessible to most forms of TMS, namely the ACC and PCC portions which lie in the interhemispheric fissure. For this reason, we decided to perform a stimulatory treatment aimed at the only part of the DMN accessible to us, which was the IPL. We utilized 200 pulses of iTBS to the right IPL, specifically the superior angular gyrus, for a total of 5 sessions. There were no complications during the sessions.

After the first treatment session, the patient began speaking single words and following simple commands. Prior to discharge after the 5 sessions were completed, this patient showed some improvement in motor function and intermittently was following commands. This enabled him to participate with some physical and speech therapy.

He returned 2 weeks later and was dramatically improved. He was able to sit up and hold detailed conversations with the neuro‐oncologist about the risks and benefits of chemotherapy for his tumor. His KPS had improved to 80, and he had begun to walk and regain some strength.

**FIGURE 2:**
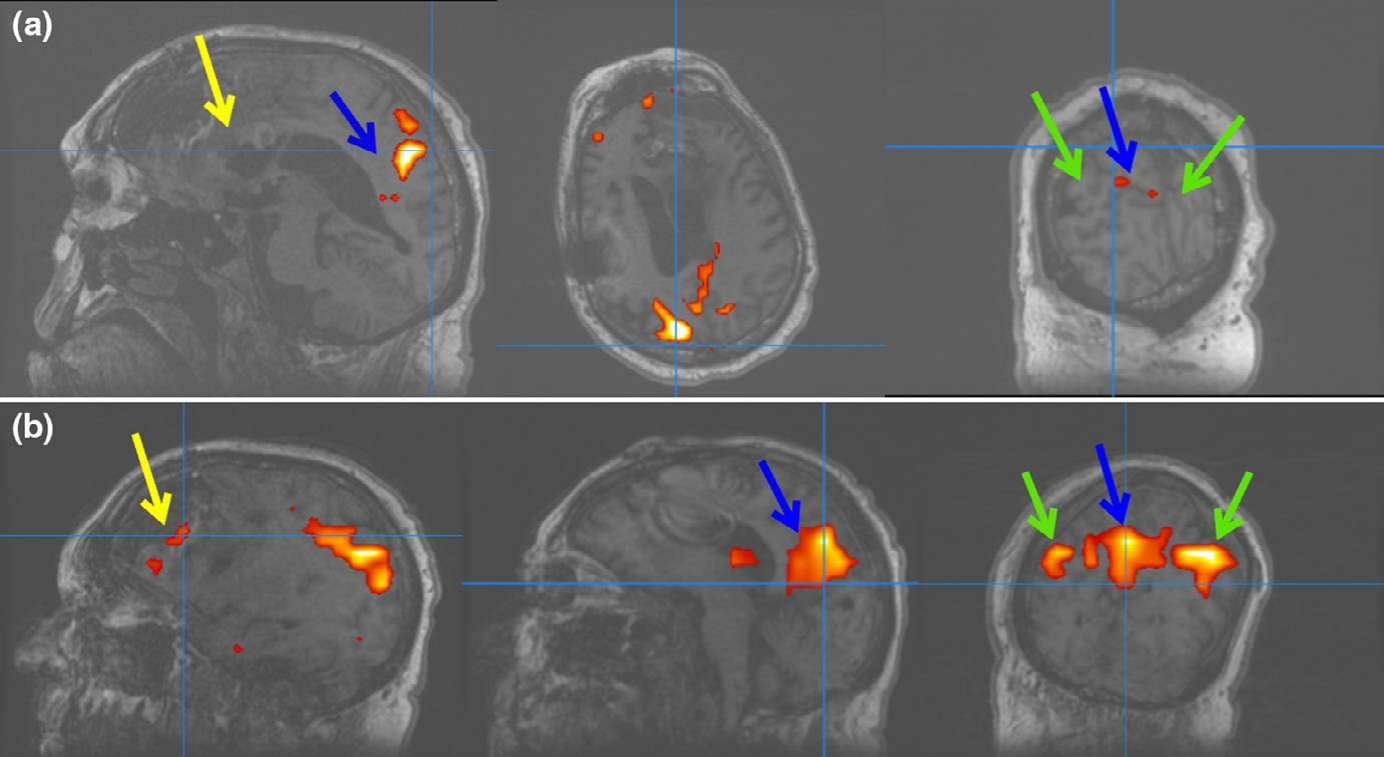
Resting‐state functional magnetic resonance imaging data demonstrating the independent component analysis (ICA) of the default mode network (DMN) in the patient (a) pretranscranial magnetic stimulation (TMS) and (b) post‐TMS. Blue arrows represent the posterior cingulate cortex, green arrows represent inferior parietal cortex, and yellow arrows represent the anterior cingulate cortex, wherein the DMN components would be expected to be seen. There was no clear evidence of the DMN in the pre‐TMS (a) ICA analysis as demonstrated by no components near the arrows. The closest component in the pre‐TMS (a) analysis near the posterior cingulate cortex seems to represent the default attention network, not the DMN. Post‐TMS (b), components representing the DMN appeared, as seen by the red areas of activity demonstrated by the arrows

We repeated a rsfMRI and were able to locate a clear DMN component in our ICA analysis (Figure 2). In addition to containing activation in the bilateral IPL's, this component contained bilateral activation in the PCC's and the right medial frontal lobe/ACC, suggesting that the DMN was a coherent independent component.

## 4 DISCUSSION

This report provides evidence that it is possible to actively promote neurorehabilitation with brain stimulation targeted at a component of the DMN in select patients. In this case, TMS was lifesaving and demonstrated dramatic functional recovery in an otherwise neurologically impaired patient. While patients do recover spontaneously from bad neurologic state, the relative length of time with minimal neurologic improvement demonstrated in this case along with timing of our patient's recovery immediately following initiation of TMS renders the idea of this being a mere *coincidence* of timing less likely than the more plausible explanation that his dramatic improvements were from the TMS treatment. Furthermore, the correlation of his improvement with the return of an independent component in the DMN regions also makes us more optimistic that the treatment was the key event driving his improvement. Importantly, in our experience with patients with frontal lobe syndromes from brain tumors, it is rare to suddenly make a dramatic recovery this far after showing no sign of improvement.

Beyond the inherent limitations relative to those posed by getting data from a single case, it is worth noting that this case does not imply that such a treatment might improve *all* patients with minimal consciousness. This patient had an injury specific to an anatomical component of the DMN and did not have any injury to the reticular activating system or other deeper structures. Thus, DMN targeted therapy might be specific to this patient and not to other patients with different pathoanatomy. Furthermore, while this patient exhibited drastic improvements in KPS scores simultaneously along with their improvement in DMN connectivity, additional and more rigorous cognitive and motor testing should be performed in future studies by a blinded reviewer to better the symptom‐specific, associated connectivity changes induced by individualized TMS treatment.

It is clear from the vast literature on the DMN that the DMN is involved with a number of important aspects of cognition (Marino et al., 2016). As these reports are mainly in either healthy patients, or less catastrophic injuries than loss of a lobe of the brain, the link between this network and more essential functions such as initiation of actions and global cognitive integration is unclear. Some evidence supports our contention that normal function of the DMN and its associated networks might be necessary to participate in life (Marino et al., 2016). For example, a lack of DMN‐control network anticorrelation has been shown to be a key feature differentiating minimally conscious from vegetative patients in a small study (Threlkeld et al., 2018). A similar network phenotype has been associated with the negative systems of schizophrenia in other studies (Hu et al., 2017). Taken with our experience with butterfly gliomas (which cross the corpus callosum), namely that avoidance of these networks during resection is necessary and sufficient for avoiding causing abulia or akinesis in these patients, the current findings build to a growing argument that in some patients, loss of these networks and their function might be a major cause of unresponsiveness (Burks et al., 2017).

It is also critical to note that loss of one part of a network might not completely destroy the network, but could indeed disorganize its oscillatory synchrony. Synchronization of distinct cortical regions is central to the formation of networks, and this report raises the idea that TMS may be an option in some patients to re‐establish this synchronization through some mechanism (Marino et al., 2016; Threlkeld et al., 2018). It is exceedingly unlikely that this patient formed new connections of substance in the time period of his recovery, so a shorter time frame mechanism is needed to explain these observations. While synaptic plasticity changes are possible, we would argue that restoration of synchrony seems more plausible. An improved synchronization of oscillations is probably the way that the DMN regions became synchronous enough to become an independent component of the wave form again.

Is reconstitution of the DMN the mechanism by which this patient improved? This obviously is very hard to conclude with certainty as likely other regions and possibly other networks were stimulated directly or transsynaptically by the DMN field. The left parietal lobe is not known for its central role in maintaining alertness, and its loss is not associated with coma. Thus, it is hard to link this to solely a direct effect. Other networks could be involved in the result. For example, parts of the control network lie in the parietal lobe, mostly anteriorly to our target (Igelstrom & Graziano, 2017). However, it is hard to believe how changing the control networks function would not involve altering the DMN to some extent, given what is currently known about the alternating interactions of these two networks (Marino et al., 2016; Threlkeld et al., 2018). Alternately, it is possible that TMS works transynaptically to change deeper, rhythm‐generating brain regions like the thalamus, or basal ganglia, with complex effects on global brain rhythms. While this is possible, given the relative segregation of thalamic inputs or outputs, cross‐talk on this scale seems hard to agree with: for example, how would the parietal lobe, which signals via the lateral thalamic nuclei, impact the posterior cingulate or contralateral targets? Finally, it is possible that TMS just resets the global set of rhythms in the recovering brain and allows the natural networks to reform. In this idea, another brain network or rhythm was also restored by giving TMS to the parietal lobe, and the return of the DMN as a coherent network was an epiphenomenon. This is of course, *possible*. While the butterfly coil we used to deliver the stimulation delivers a relatively focal field, TMS is notoriously broad in what it stimulates directly and indirectly. Thus, by itself, this observation cannot definitively say that in this patient, stimulating the angular gyrus with iTBS had a remarkable result.

Regardless of how the treatment actually worked, this case raises questions about the future of individualized neurorehabilitation. While this has generally meant some variation on physical, occupational, or speech therapy, completed away from the treating clinicians who managed the initial insults, this raises the idea that perhaps we should reconsider this model. It seems reasonable to hypothesize that if TMS can potentiate rehabilitation, then we should be getting this treatment to more patients to test this hypothesis. It is without question that neurologic hospitals are full of people with little to lose by trying TMS, and that by not considering it as part of rehabilitation, we may be denying some patients more effective rehab. Furthermore, it is possible that initiation of earlier therapy might be more effective. The idea that there are akinetic mute patients who may be able to return to some quality of life with a noninvasive low‐risk treatment that we are not attempting to deliver to them is a haunting prospect.

In addition, we argue that given new insights in connectomics, biophysical modeling, and advanced imaging, that there are several new questions to be addressed in the brain function and recovery of the critically neurologically ill, and the better ability to target our treatments to the patient's actual problem and to predict what is likely to happen with our therapies, at least in the immediate term.

In short, while this admittedly is a single case, it is a dramatic and provocative one which raises numerous neuroscientific, clinical, and ethical questions about how we should be rehabilitating patients with brain injury. It principally asks the provocative question about whether losing one's DMN is akin to losing one's will.

## CONFLICT OF INTEREST

Michael Sughrue is the Chief Medical Officer, and Charles Teo is a cofounder of Omniscient Neurotechnologies. No products related to this were discussed in this paper. No other authors report any conflict of interest.

## AUTHOR CONTRIBUTIONS

**TressieM. Stephens** involved in formal analysis, investigation, and writing—original draft. **Isabella M. Young** involved in writing—original draft and validation. **Christen M. O'Neal** involved in methodology. **Nicholas B**. **Dadario** involved in writing—review and editing. **Robert G. Briggs** involved in data curation and resources. **Charles** **Teo** involved in supervision. **Michael E**. **Sughrue** involved in conceptualization, methodology, and supervision.

### PEER REVIEW

The peer review history for this article is available at https://publons.com/publon/10.1002/brb3.2180.

## Data Availability

The data that support the findings of this study are available from the corresponding author upon reasonable request.
